# New Insight into Bacterial Interaction with the Matrix of Plant-Based Fermented Foods

**DOI:** 10.3390/foods10071603

**Published:** 2021-07-10

**Authors:** Klaudia Gustaw, Iwona Niedźwiedź, Kamila Rachwał, Magdalena Polak-Berecka

**Affiliations:** Department of Biotechnology, Microbiology and Human Nutrition, Faculty of Food Science and Biotechnology, University of Life Sciences in Lublin, 8 Skromna Street, 20-704 Lublin, Poland; klaudia.gustaw@up.lublin.pl (K.G.); koprukowniak.i@gmail.com (I.N.); magdalena.polak-berecka@up.lublin.pl (M.P.-B.)

**Keywords:** plant fermentation, lactic acid bacteria, bacterial community, health benefit, bioactive molecules, probiotics, functional foods, food microbiology

## Abstract

Microorganisms have been harnessed to process raw plants into fermented foods. The adaptation to a variety of plant environments has resulted in a nearly inseparable association between the bacterial species and the plant with a characteristic chemical profile. Lactic acid bacteria, which are known for their ability to adapt to nutrient-rich niches, have altered their genomes to dominate specific habitats through gene loss or gain. Molecular biology approaches provide a deep insight into the evolutionary process in many bacteria and their adaptation to colonize the plant matrix. Knowledge of the adaptive characteristics of microorganisms facilitates an efficient use thereof in fermentation to achieve desired final product properties. With their ability to acidify the environment and degrade plant compounds enzymatically, bacteria can modify the textural and organoleptic properties of the product and increase the bioavailability of plant matrix components. This article describes selected microorganisms and their competitive survival and adaptation in fermented fruit and vegetable environments. Beneficial changes in the plant matrix caused by microbial activity and their beneficial potential for human health are discussed as well.

## 1. Introduction

The fermentation process has been known and used since ancient times, but the fermentation techniques developed thousands of years ago are still being improved and investigated. Fermented vegetable products are very popular and are produced all over the world. In some countries, they are an important component of the daily diet. The process of fermentation of plant substrates is carried out by microorganisms (bacteria, yeast, and molds) involved in the transformation of components present in the plant matrix [1,2]. During the fermentation process, organic matter is converted into energy by enzymes produced by living microorganisms. In the food context, fermentation consists in conversion of food raw materials into non-toxic preserved products, which often exhibit health-promoting properties [3]. In turn, microorganisms change their living environment by metabolizing substrates and converting complex molecules into simpler compounds and nutrients. Various microorganisms are involved in the fermentation of plant-based raw materials [4]. Microorganisms, typically bacteria and yeasts, coexist and interact with the environment either spontaneously or through a controlled fermentation process. Over the past 20 years, the genomes of various strains have been sequenced, often in combined transcriptome or metagenomic studies, which led to the elucidation of the key aspects of the potential mechanisms associated with bacterial adaptation to plant-based food as an ecological niche [5]. The growing number of strains and research on new fermented products have shed a light on the adaptive potential, different mechanisms, and progressive evolutionary processes of microbial communities [6]. Currently, new strains with interesting properties that can improve products are constantly being investigated. In particular, *Lactobacillus* bacteria are the main focus of interest, as they improve the sensory properties, consumer attractiveness, and chemical composition of food. Particularly important and interesting is the generation of compounds with biological activity resulting from microbial transformations of plant material. Thus, microbial fermentation can be regarded as a potential technology for the release of certain bioactive compounds from natural resources [7]. Simultaneously, fermentation processes involve initial digestion of foods to improve absorption thereof in the human body [8]. Moreover, they enrich food with such ingredients as proteins, sugars, vitamins, and amino acids. In addition to nutrients, microbial fermentation of plant material often yields bioactive compounds with a beneficial effect on human health, e.g., phenolic compounds, phytosterols, and vitamins, which can counteract the development of certain diseases [9,10,11].

For a long time, fermented vegetables have been arousing considerable interest due to their health benefits. Fermented foods and plant-based beverages are products with high nutritional and functional value and a positive effect on human health. They can be a natural sustainable alternative counteracting the high wastage of fruits and vegetables due to their short shelf life [2]. Moreover, the demand for plant-based alternatives to fermented dairy products has been increasing in recent years. Simultaneously, meeting consumer expectations is a considerable challenge for food producers. Fermentation is a key process in the transformation of plant-based raw materials into non-dairy alternatives [4]. The demand for plant-based protein sources as a substitute for meat is growing due to the sustainability and human health concerns and for ethical reasons. Traditional plant-based meat substitutes (e.g., tofu or seitan) are not very popular in Western societies, while such meat analogues as textured plant proteins are gaining popularity. Another example is kombucha, i.e., a non-alcoholic fermented beverage with a comparable flavor but no alcoholic side effects offering obvious health benefits. With the increasing consumer awareness, fermented plant-based foods are becoming a food trend. Fermentation provides a product with a satisfactory shelf life, interesting flavor, and beneficial health properties.

The transformation of plant material during fermentation results in changes in the organoleptic and nutritional properties of the product. It increases food safety, extends the shelf life, and provides certain nutritional and sensory values [12]. The effect of fermentation on the plant matrix is specific to the microorganisms involved and depends on the plant material used, especially its unique chemical composition. Moreover, it can be modified by exogenous environmental conditions of fermentation. In this article, we focus on recent research of bacterial effects on fermented foods of plant origin. Identification of the effects of microorganisms on the plant matrix is critical to understanding how to manipulate the process and the strains used for inoculation to achieve the desired results [13].

## 2. Literature Review

### 2.1. Molecular Adaptation of Microorganisms to the Plant Fermentation Niche

Over the centuries, humankind has discovered and managed to master the extremely complex process of fermentation in which only beneficial microorganisms protecting food from spoilage are allowed to act. Many microorganisms colonize the surface of raw fruits, vegetables, and cereals. They are mainly represented by aerobic gram-negative bacteria as well as yeasts and molds. Lactic acid bacteria are only a small part of the primary community. *Weissella cibaria/confusa* and *Lactiplantibacillus plantarum* are the most common species [14]. Most plants, vegetables, and fruits can undergo spontaneous lactic fermentation if appropriate conditions are provided, i.e., no access to oxygen, proper osmotic conditions—humidity and salt concentration, and moderate temperature, which are all suitable for promoting the growth of lactic acid bacteria. Other microorganisms, i.e., gram-negative bacteria, yeasts, and molds, are suppressed by the addition of salt in the initial stage of fermentation and then by various mechanisms, including the production of organic acids by LAB.

Due to their homofermentative metabolism, bacteria of the genus *Lactococcus* are better producers of lactic acid than heterofermentative bacteria. The latter type of metabolism, e.g., in *Leuconostoc* and *Lactobacillus*, imparts complex organoleptic properties by shifting the reaction towards the production of other metabolites, organic acids, and volatile compounds [15]. Bacteria of the former genus *Lactobacillus* dominate, as they occupy the niche of fermented products in the final stages [16].

The microorganisms listed above usually have unique functional properties. It seems that the desired end result, i.e., a preserved plant-based product, is the outcome of a series of adaptations of these microorganisms to claim the niche of the raw plant material. The basis of LAB metabolism is their ability to utilize available plant nutrients rapidly. The diversity of vegetable and fruit ecosystems has resulted in the evolutionary binding of the microorganism to its living environment. Due to loss and/or acquisition of new genes, lactic acid bacteria have adapted to a number of compounds present in their niches. These environments provide growth promoting agents and essential carbohydrates but create conditions that inhibit bacterial growth, e.g., the presence of phenolic compounds, unfavorable osmotic pressure, non-standard energy sources, non-fermentable carbohydrates, and physical factors. Although the experimental approach of culturing isolated microorganisms is still considered acceptable, the omics approach is currently gaining supremacy in investigations of fermentation microbiota. Molecular techniques help to circumvent the limitations associated with laboratory non-culturable microbial communities and reflect true events and adaptation in studies of a collection of various microorganisms interacting with each other and with environmental factors rather than individual species in a laboratory-prepared medium. In this subsection of the article, we intend to focus on a coherent description of the latest findings of the molecular basis of bacterial adaptation to plant-based fermented foods. The comprehensive genome analysis of an individual strain and the metagenomic approach investigate the source of microbial adaptation to the fermented plant product niche based on gene comparison and annotation, clustering, and phylogenetic studies. In response to external signals, LAB down-regulate the expression of genes associated with central metabolism, while activating alternative pathways, transport of metabolites, and responses associated with functional traits relative to the plant matrix. To a lesser extent, lactic acid bacteria make use of genes that are generally assigned to stress responses [17]. An overview of recent molecular genomic publications and details of strain adaptation to the fermented plant-based food environment are presented below.

The overview starts with *L. plantarum* as the most “nomadic” species. Since it has one of the largest genomes (approximately 3.5 Mb according to NCBI sequences 2.91 to 5.34 Mb), the species is known for its adaptations to a variety of environments, including fermented foods (from sauerkraut, pickled corn, cucumbers, radishes, bananas, oranges, and grasses to meat, milk, wine, and the digestive system). Considering the relationship between evolution and adaptation to the environment, for unclear reasons, *L. plantarum* has retained the “unnecessary genes”, and the genome has become “universal” and independent of the environment. Each *L. plantarum* strain has an individual repertoire of genes associated with the transport and utilization of sugars, corresponding to the carbohydrate composition of a fresh and fermented vegetable or fruit. Genes responsible for sugar metabolism are accumulated together in the genome (so-called island) [18]. Particularly important in terms of adaptation to plant material utilization is the ability to degrade complex carbohydrates such as amylose and starch. Moreover, in order to make fermentation substrates available for use, in strains that utilize simple sugars, i.e., glucose, fructose, and sucrose present, e.g., in cabbage and radish, require transport systems. In the case of another species, i.e., *Latilactobacillus sakei*, the ability to conduct a homo- and heterolactic fermentation process can be considered an adaptation, whereas *Leucnostoc mesenteroides* and *Weissela koreensis* involved in kimchi fermentation are exclusively heterolactic organisms [19,20]. Furthermore, *L. sakei* can metabolize a wider range of carbohydrates than the kimchi-LAB, which is advantageous in the later stages of fermentation when primary sugars have already been utilized [21]. Sourdough isolates of *Limosilactobacillus fermentum* have genes responsible for degradation of the major carbohydrates: starch, maltodextrin, maltose, and sucrose, conversion of fructose into mannitol, and production of acetoin and L-ornithine. All these features are beneficial for sourdough fermentation [22]. *Latilactobacillus curvatus* strains isolated from pickled radishes/carrots, kimchi, and baechu (chinese kimchi) have been examined. The strains exhibited a rich repertoire of carbohydrate metabolism and associated PTS systems, compared to strains isolated from meat. *L. curvatus* strains showed convergence in the catabolism of carbohydrates present in plants, such as glucans, fructans, and acetylglucans [23]. The niche-dependent clustering in this species, in contrast to the lack of genome niche-dependence in *L. plantarum*, suggests that the greatest similarity between strains and the ecological niche should be sought within the repertoire of genes encoding metabolism of compounds present in the niche.

Three species of the genus *Lactococcus* have been included in the microbial food cultures (MFC) inventory: *Lc. lactis* subsp. *lactis*, *Lc. cremoris*, and *Lc. raffinolactis* are often used as starter cultures for food production [24,25]. It has been shown that the frequent use of these strains as starter cultures has induced changes in their genomes dividing the strains of this genus into “domesticated” and “environmental” strains. The latter exhibit a huge diversity of adaptive mechanisms and richer genomic repartition; hence, they are able to colonize not only fermented foods of plant origin but also more inaccessible environments such as living plants, e.g., grass or sugar cane [26,27]. Strains associated with a plant niche encode a wider range of metabolic pathways than dairy strains, since lactose is the main carbon source in milk, while each plant niche has an individual carbohydrate composition. Moreover, genes involved in pentose and glucurate metabolism are virtually exclusive to the genomes of bacteria associated with the plant niche. Similar to KF147, *Lc. lactis* subsp. *lactis* strains have a pathway related to the metabolism of plant sugars raffinose and arabinose [26,28]. Multiple glycoside degradation pathways have been demonstrated in the KF14 isolate; the strain also uses pathways to metabolize hemicellulose, cellobiose, xylose, and arabinose characteristic of plant habitats. This genus also possesses genes responsible for biofilm formation (exopolysaccharides), which is crucial for survival on the plant surface, and for production of bacteriocins, which to some extent increase the lifespan of *Lactoccous* strains during fermentation, as they dominate in the initial stages of fermentation due to their sensitivity to low pH [29]. Environmental adaptation traits are often plasmid-encoded, and the genus often has several plasmids encoding 4–8% of the genetic information, functional properties such as lactose fermentation, protease activity, and resistance to bacteriophages [26,30].

The environment of fermented fruits is characterized by high sugar concentrations, which induce the evolution of another strategy—fructophilicity in the non-phylogenetic group of fructophilic lactic acid bacteria (FLAB), which prefer fructose versus glucose as their main carbon source [31]. Their adaptation to the environment resulted in an extremely reduced genome. While *Lactobacillus* spp. have an average genome size of 2.5 Mbp, *Apilactobacillus micheneri* has a genome size of 1.42 Mbp. FLAB need an external electron acceptor to balance NAD/NADH; due to the lack of the bifunctional alcohol/acetaldehyde dehydrogenase adhE gene, they do not have a complete respiratory pathway [32]. Through adaptation to the plant niche, the genera described above (*L. plantarum*, *L. sekei*, *Lc.* spp.) have retained or even increased the gene pool associated with carbohydrate utilization; the exact opposite is true for FLUB. Comparison of FLAB genomes revealed common deficiencies in carbohydrate metabolism and transport pathways rendering these bacteria incapable of utilizing galactose or mannose [33]. Noteworthy, despite the reductive evolution of the fructophilic genomes, the bacteria are remarkably well adapted to the high concentrations of simple sugars: glucose and fructose, which are prevalent in the fermented fruit environment [34,35]. A unique type of convergent adaptation is observed in FLAB.

*Leuconostoc mesenteroides* is one of the major LAB species used in kimchi or cabbage fermentation. These bacteria are present in the early stages of the process, start fermentation probably due to their ability to grow at relatively low temperatures, and die due to their sensitivity to the increasing environmental acidification. The latter trait is a weakness of the genus. It has been demonstrated that a mutation in the atpC gene and overexpression of F0F1 ATPase in these bacteria may cancel the lethal effects of this environmental factor [36]. A strong strain-dependent association of environmental adaptation with carbohydrate metabolism was also noted in *Ln. mesenteroides*. This is confirmed by the distribution of genes from the category of COGs, with approximately 10% of unique genes and only 4% of core genes [19]. In addition, mannitol dehydrogenase, which converts fructose present in early stages of kimchi or sauerkraut fermentation, was detected in all strains as well. This feature increases the competitiveness of this species, as mannitol production is associated with ATP production, compensating for the differences in the energy between heterofermentative *Leuconostoc* and other homofermentative LAB. Transcriptomic studies have also revealed that glucose, fructose, and mannose are most abundant in the kimchi environment, which is reflected by high expression; however, in contrast to the FLAB described above, *Leuconostoc* spp. prefer glucose. Glucose transporters are more frequently used in the initial stages of fermentation. Probably, after depletion of this sugar, the expression of fructose, mannose, trehalose, or sucrose transporters increases. However, noteworthy is *Leuconostoc citreum* F192-5, i.e., a fructose-preferring strain of this genus adapted to sugar-rich environments and isolated from satsuma mandarin peel. No progressive reduction of the genome, especially genes responsible for carbohydrate metabolism, was observed in this “pseudofructophilic” strain [37]. The exceptional existence of this strain seems to confirm and explain the molecular adaptation determining the differences between these genera.

Since glucose is readily available and used by most microorganisms during the initial stages of fermentation, once it is depleted, bacteria that efficiently utilize another carbon source gain advantage. Bacteria of the genus *Weissela* dominate during later stages of kimchi fermentation over *Ln. mesenteroides*, which utilize glucose deposits early in the fermentation process. Ribose transporter genes are transcribed more intensively in *W. koreensis*, as in the case of mannose, arabinose, and gluconate, while the expression of the glucose transporter is relatively low. The competitive advantage of *W. koreensis* to survive during kimchi fermentation is based on the use of the galacturonate metabolism pathway, as this compound is part of the pectin-component of the plant cell wall [20].

The genus *Enterococcus* has representatives that participate in plant fermentation as well as opportunistic and even pathogenic strains. *Enterococcus faecium* strains from traditional Korean soybean-based products (Doenjang, Ganjang, Meju) were aligned with 51 pathogenic or opportunistic strains and 52 strains of different origin. It was revealed that, despite the phylogenetic relatedness, nonclinical strains in the hierarchical analysis were assigned to other clusters. Strains isolated from fermented soy products did not have genes related to virulence factors, genes responsible for antibiotic resistance, and mobile elements. To adapt to the soybean niche and, in particular, to the abundance of soybean proteins in soybean, *E. faecium* strains carry genes related to sugar metabolism (mannose/fructose cluster) supported by other genes, including PTS transporters and permeases. It is likely that *Bacillus* bacteria are involved in the breakdown of complex compounds during fermentation of soybeans, and the products are then metabolized by *E. faecium* [38]. The most significant studies that have been conducted to date on molecular characteristics responsible for bacterial adaptation to the plant-based fermented foods are summarized in Table 1.

Adaptation to the environment of fermented plant products certainly plays an integral role in shaping the content of the genome. A clear classification of strains in terms of genome load is not evident in all the genera described, but the reductive evolution of genomes is widely observed. The linkage between the environment and adaptation to a specific niche has been observed. Genes responsible for carbohydrate metabolism demonstrate apparent grouping of organisms relative to the niche in which they occur. This suggests the relevance of carbohydrate metabolism genes as both a strain-dependent trait and the need for some minimum collective repertoire. The plant environment is considered less nutritionally rich compared to dairy products, which is probably the reason why the number of genes included in the metabolic category is so abundant in so many species. The mechanism of decomposition and transport of carbohydrates to some extent shapes the lifestyle of the bacterium, the choice of a preferred source of carbon, or the ability to use unusual carbohydrates determining survival in the environment of plant origin, while such features as production of exopolysaccharides and bacteriocins play a secondary role. A model has been described in *L. plantarum* where the universal genome does not limit this bacterium to one niche or induces far-reaching reduction and specialization towards one sugar, fructose or rhamnose, yielding a mono-associated bacterium.

### 2.2. Physicochemical and Biological Properties of Fermented Plant Material

**Figure 1:**
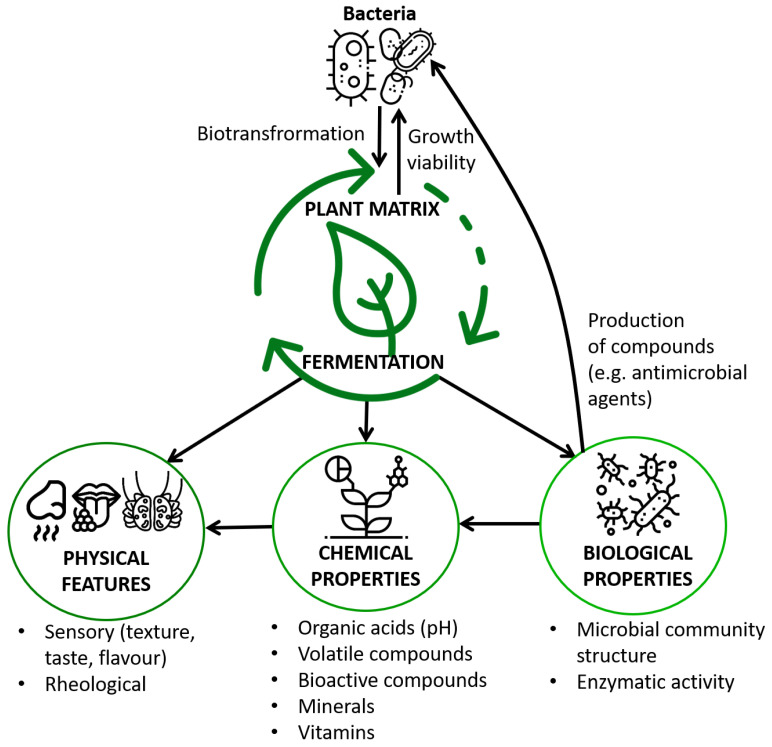
Fermentation as a determinant of the physical, chemical, and biological properties of plant material.

During fermentation, the plant matrix undergoes certain modifications. The physical properties of the plant material may change after fermentation and these changes may affect the biological and organoleptic properties of fermented food products. Initial characteristics of plant substrates used for fermentation may vary considerably in terms of their chemical composition and biological properties. This also influences the physiochemical and biological composition of the resulting product (Figure 1). The impact of the fermentation process on the plant matrix is dependent on the material and specific to the fermentation microorganisms. It is therefore possible to modify the final result by modifying the external environmental conditions of the process. Many product parameters can be controlled in order to obtain a product with attractive properties for the consumer. Not only the functional attributes but also such organoleptic features as the flavor, smell, and color are important. The pH value, fatty acid profile, mole solution, oxygen, humidity, duration of fermentation, remodeling of organic matter, color, texture, and rheological properties (e.g., consistency, stickiness, hardness, viscosity, and adhesiveness) are physicochemical properties that may change during fermentation. The changes are a result of biochemical processes occurring during fermentation and changes in oxygen and temperature. Changes in the physical properties of the product induced by fermentation influence the chemical and sensory characteristics of the fermented food [44]. These processes also have an impact on the biological properties and the population of bacteria present in the product, i.e., the number of bacteria and the composition of bacterial community may be altered.

The pH value of a fermented product is an important factor closely related to the activity of microorganisms involved in fermentation. It contributes to microbiological stability against pathogenic and spoilage bacteria and is related to the flavor of the product. This was observed, e.g., in the analysis of a fermented camu-camu and soymilk combination. The reduction in the pH value was more rapid during fermentation carried out by *L. plantarum* than in the presence of *Lactobacillus helveticus*. This is related to the ability of particular bacterial strains to produce organic acids [45]. These compounds are generated in the process of decomposition of organic matter by microbes. The presence of organic acids, especially lactic acid, reduces the pH value to 5.0 or less [46]. The mechanism of this phenomenon is based on acid dissociation resulting in the release of hydrogen ions, which changes the balance of the solution and decreases the pH. For instance, it has been observed that fermentation of white beans by lactobacilli reduces the pH to 3.7–4.7 [47]. *L. plantarum* CCMA 0744, *L. fermentum* CCMA 0745, and *Lc. lactis* CCMA 0415 used for yam (*Dioscorea* spp. L.) fermentation contributed to a decrease in pH from 6.1 to 3.7–3.8 through lactic acid production [48]. Acidification of fermented soy beverages to pH 3.5 after 48-h fermentation by lactobacilli at 37 °C was observed [49]. In the case of kimchi (fermented Chinese cabbage), the pH decreased from 5.34 observed in the early stage of fermentation to 4.30–4.40, which was maintained on the subsequent days of storage (7–67 days). This pH value was related to the presence of lactic and nitric acids [50]. Other authors have shown a decrease in pH from a value of 5.0–5.4 at the beginning of fermentation of kimchi to 4.0 after 57 days of fermentation [51]. In turn, the pH value during fermentation of pineapple increased from 3.4 to 4.0 for fruits fermented by *W. cibaria* and to 3.5 for those fermented by *Ln. pseudomesenteroides*, and the value remained constant after 16 days of storage. These changes may be caused by decarboxylation of citric or malic acid, which are present in pineapple [43]. Similar data were obtained after fermentation of prickly pear (*Opuntia ficus-indica* L.) fruit puree by LAB. The pH value decreased to approx. 3.92–4.10 during storage and further to ca. 3.72–3.78 after 21 days, which was connected with the production of lactic and acetic acids by the bacteria [52].

A low pH not only results from the presence of microorganisms but also affects their community. At a low pH, lactic acid bacteria begin to dominate in the product. In the later phase of fermentation of plant material in acidic conditions, organic acids and protein are decomposed, resulting in release of carbonic acid, ammonia, and a small amount of N_2_, CO_2_, and CH_4_, thus increasing the pH value again [46]. The growth of microorganisms during fermentation is also closely related to temperature. The activity of microorganisms in the fermented product contributes to an increase in the temperature of the plant material, since energy is generated in the form of heat, CO_2_, and water vapor during the decomposition of organic matter. After reaching a maximum value, the temperature during fermentation begins to decrease, possibly as a result of the activity of microorganisms contributing to the reduction in the nitrogen content of the material as they break down organic matter into simpler compounds. Nitrogen and organic material are used by microorganisms for their activity and development; therefore, a decrease in their content reduces the number of microorganisms in the product [53].

The structure of microbial communities is an important factor influencing the fermentation process. The initiation and progression of fermentation depends on the bacterial microbiota. Bacterial growth during fermentation has been shown to vary depending on the components of the plant material, storage method and temperature, salinity of the product, etc. The type of plant material can promote the growth of certain bacteria during fermentation. This was demonstrated by Fujita et al. in a study on a fermented product containing a combination of soymilk with camu-camu powder. Both *L. plantarum* and *L. helveticus* were able to grow successfully in this plant material, but *L. helveticus* had better performance of growth and stability during fermentation. This was apparently related to the high protein content in soymilk, which contributed to the growth of *L. helveticus*, a known protease producer [45]. Fermented soy was found to support the growth of BB-12^®®^ *Bifidobacterium*. Furthermore, the viability of this strain was demonstrated in soy desserts during six months of storage (viable cell count > 7 log10 CFU/g). Studies comparing different plant matrices showed that apple juice was a favorable environment for the development of *Lactobacillus* spp., while orange juice had an adverse effect on the bacteria. It was also observed that fermented cherry or pineapple juices were not a good environment for the growth of *L. plantarum*, in contrast to vegetable juices, e.g., from tomatoes and carrots [54]. Pineapple juice, in turn, was indicated as a suitable environment for the growth of *W. cibaria* and *Ln. pseudomesenteroides*. The number of these LAB during pineapple juice fermentation increased from 1.0 × 10^5^ CFU/mL (upon inoculation) to 7.5 × 10^5^ CFU/mL and 7.5 × 10^7^ CFU/mL for *W. cibaria* and *Ln. pseudomesenteroides*, respectively. During refrigerated storage of the fermented product, a decrease to 1.5 × 10^5^ CFU/mL and an increase to 1.6 × 10^8^ CFU/mL were recorded in *W. cibaria* and *Ln. pseudomesenteroides*, respectively [55]. In turn, a non-dairy yogurt-like product obtained by fermentation of almond milk turned out to be a favorable environment for the growth of such probiotic bacteria as *Limosilactobacillus reuteri* and *Streptococcus thermophilus*. The viability of these bacteria in the product declined over 28 days of cold storage. Nonetheless, the abundance of *L. reuteri* was retained at approx. 7 log10 CFU/mL [10]. In another study, *L. lactis* CCMA was shown to be a suitable starter culture for yam fermentation. During fermentation, this strain maintained approximately 8 log10 CFU/mL cell viability [48]. A study conducted by Jung et al. on kimchi showed changes in the total bacterial abundance in the plant material during fermentation and storage of the product [50]. The bacterial counts increased with the fermentation time. This was particularly noticeable in the case of *Lactobacillus*. *Lactobacillus plantarum* are the main species causing acidification of the product during fermentation. Their number increases as the number of *Ln. mesenteroides* decreases. The changes in the bacterial population during fermentation have been well studied in fermented bread sourdough. Regardless of the type of flour used, characteristic dynamics in the bacterial population present in the sourdough was observed. Initially, species belonging to the genera *Lactococcus*, *Enterococcus*, and *Leuconostoc* dominated in the sourdough, followed by LAB species from the genera *Pediococcus*, *Lactobacillus*, and *Weissella*. A predominance of heterofermentative species *L. plantarum.*, *L. fermentum*, *Fructilactobacillus sanfranciscensis*, and *Limosilactobacillus ponti* was noted as well [56]. Changes in the microbial community during fermentation of plant products may additionally be caused by the production of antibiotics by microorganisms, including bacteriocins, which inhibit the growth of harmful bacteria [50]. Microbial analysis of kimchi carried out by Maoloni et al. revealed formation of an active microbial community consisting predominantly of mesophilic aerobes and lactic acid bacteria [51]. The study showed that the total number of these bacteria in the product after nine days of fermentation reached >8 log10 CFU/g. The number of LAB gradually increased until the 36th day of fermentation, when it achieved a maximum value of 6 log10 CFU/g. In contrast, a significantly higher number of viable *Lactobacillus* cells, i.e., approx. 9 log10 CFU/g, was shown in previous studies on the microbial dynamics in naturally fermented kimchi. At the initial stages of kimchi fermentation, *Enterobacteriaceae* and *Pseudomonadaceae* were also present in the product, and their number decreased during the fermentation. At the early stages of the process (first 15 days), *Erwinia* spp., *Pseudomonas viridiflava*, *Pseudomonas veronii*, *Sphingomonas* spp., and *Rahnella aquatilis* were identified in the kimchi. In the subsequent stages (between days 15 and 36), there was a significant change in the bacterial community structure in the kimchi. The spoilage species were almost completely replaced by lactic acid bacteria. At this stage, *Leuconostoc kimchi* and *Weissella soli* were the most abundant bacteria present in the product. These changes were related to the accumulation of organic acids produced by LAB, which create unfavorable environmental conditions for the existence of undesirable bacteria [51]. Research conducted on a bean-based beverage extracted from germinated seeds of white bean cv. “Piękny Jaś Karłowy” (*Phaseolus vulgaris*) showed that this plant substrate promoted the activity of lactobacilli. The live cell population of these bacteria in the product was above 7 log10 CFU/g. The microbial community was also studied in fermented cakes made from sorghum flour with oat and rice bran. In the bran and sorghum flour dough, a large number of *Enterobacteriaceae* cells were found during fermentation. In the control dough containing buckwheat bran, the number of *Enterobacteriaceae* was at the level of 4 log10 CFU/g after one day of fermentation, but reached approx. 5 log10 CFU/g at the end of the process. In the control dough made from rice bran and oat bran, the density of these bacterial cells increased to more than 7.0 log10 CFU/g, although no *Enterobacteriaceae* cells were present in the dough before fermentation. Inoculation of these products with *L. brevis* cells reduced the *Enterobacteriaceae* cell counts by at least 1 log unit, compared to control samples. The *Enterobacteriaceae* growth was significantly inhibited when the LAB cell density increased during fermentation [39,57].

During fermentation, microbial activity changes the chemical composition of the fermented plant material [13]. Bacterium-specific metabolic features combined with plant enzyme activity can improve the bioavailability of certain phytochemicals [17]. Raw material components are enzymatically and chemically decomposed and subsequently modified in biotransformation reactions [44]. Bacteria are capable of converting substances contained in plant substrates into a variety of compounds, which can lead to a marked increase in the amount of functional microbial metabolites, often exhibiting valuable nutritional properties [13,58]. Changes in the chemical composition of plant material are mainly caused by decomposition by bacterial enzymes. Various studies have shown alterations in bacterial enzyme activity during fermentation. They were observed, e.g., during fermentation of a camu-camu and soymilk combination by LAB strains. The results showed higher inhibitory activity of α-amylase and α-glucosidase [45]. Furthermore, α-galactosidase activity has been demonstrated in soybeans fermented by the BB-12^®®^ *Bifidobacterium* strain. Its presence may reduce the galactooligosaccharide content of soymilk, which is important because these molecules cannot be digested in the human intestine [59]. Phytase is another beneficial enzyme produced by bacteria in fermented plant products. It is responsible for decomposition of phytate, which is an anti-nutritional compound. Its presence was demonstrated in, e.g., fermented quinoa sourdough. The phytase activity in this product was around 2.75-times higher than that in raw quinoa flour [60]. Fermentation of sourdough made from legume or pseudocereal flour reduced the phytate content in the final product due to the activity of cereal phytase supported by the low pH value resulting from the activity of microorganisms [13]. Phytase was detected in yams fermented with *Lc. lactis* CCMA 0415. The fermentation of yams by this strain resulted in 82% reduction in the phytate content. This bacterium also produces α-amylase during yam fermentation. The activity of this enzyme results in degradation of starch contained in the plant material to fermentable carbohydrates, which are then used by the bacteria to produce, e.g., organic acids [48]. Lactobacilli also produce fatty acid hydratase and are thus capable of converting polyunsaturated fatty acids to hydroxyl and conjugated fatty acids, which are known bioactive compounds. Linoleic isomerase activity, in turn, is related to the presence of *L. plantarum* in the fermented substrate. As a result, fermentation of certain plant materials (e.g., sunflower and castor oil or nuts) by this bacterial species results in enrichment of the product with conjugated linoleic acid [17,61]. It has been shown that, during cocoa fermentation, lactic acid bacteria can produce citrate lyase; however, its activity is strongly influenced by environmental conditions (pH, temperature). A consequence of the activity of this enzyme in the early stage of cocoa bean fermentation is the decomposition of citric acid by the bacteria to produce acetic and lactic acid [62]. In another study, Filannino et al. reported that the action of bacterial glycosyl hydrolases during lactic fermentation of cactus cladode pulp resulted in the release of two flavonoid aglycones (isorhamnetin and kaempferol) with antioxidant activities [63]. The increase in the phenolic content in products fermented by LAB is presumably associated with the action of enzymes that cause depolymerization or hydrolysis of phenolic compounds. Several enzymes, e.g., tannase and β-glucosidase (detected in *Ln. mesenteroides*, *Weisella paramesenteroides*, and *Ln. fallax*) or feruloyl esterase (observed in *Leuconostoc* spp.) can be involved in these reactions, inducing changes in the plant matrix, e.g., during fermentation of fruit juices [43]. Phenolic compounds can also be formed during fermentation as a result of tannin degradation, as demonstrated for fermented quinoa sourdough. The activity of such enzymes as oxygenases and decarboxylases or other LAB and quinoa endogenous enzymes may be involved in the degradation of tannins [60]. Fermentation of quinoa flour by lactic acid bacteria was also shown to cause proteolysis of native quinoa proteins, resulting in the release of antioxidant peptides [64].

The impact of microbial cultures on plant raw materials, with particular emphasis on their potential to improve the sensory and textural properties of fermented plant products, is a highly interesting research topic. Fermentation can result in changes in the texture and appearance of a unique aroma or flavor. The activity of microorganisms during fermentation influences the profile of volatile compounds in plant foods and beverages. In turn, these compounds contribute to the unique flavor of fermented foods. These volatile compounds, i.e., acids, alcohols, esters, ketones, aldehydes, alkanes, and others, differ in their chemical structure. It was demonstrated that yam fermentation by LAB (*L. lactis* CCMA 0415, *L. plantarum* CCMA 0744, and *L. fermentum* CCMA 0745) resulted in the production of acetoin, acetic acid, hexadecane, and 2-tridecanone, which were not present in the unfermented plant. The conversion of citric acid by LAB can lead to the production of acetic acid and acetoin, which contribute to the butter and cream flavors in products. During yam fermentation, the bacteria also produced volatile alcohols (such as 1-dodecanol, 1-tetradecanol, and hexyldodecanol), the formation of which was associated with amino acid degradation reactions [48]. In Khorasan-based foods fermented by *L. plantarum* strains with production of volatile compounds (such as carbonyls, alcohols, 1,3-hexadiene, and dodecanoic acid), the functional and sensory profile was significantly enhanced [7]. Lactic fermentation with selected *L. plantarum* strains was shown to improve the flavor profile of processed pomegranate juice. This was associated with an increase in the concentration of desirable compounds responsible for positive sensory attributes (e.g., ketones, alcohols, and terpenes). In contrast, the concentration of undesirable aldehydes, sulfur compounds, and furans, which are responsible for unpleasant odors, decreased during the fermentation. The fermented pomegranate juice was mainly characterized by higher intensity of floral, fruity, and anise flavor than the control (raw fruit), which had a lower level of volatile organic compounds resulting in low aroma intensity [65]. Changes in product sensory properties were also reported in analysis of pineapple juices fermented by LAB. Pineapple juice fermented by *W. cibaria* or *Ln. pseudomesenteroides* was characterized by a more pronounced sweet and sour taste as well as smoothness, fluidity, and freshness. In contrast to the unfermented product, the juice fermented by *W. cibaria* was characterized by a yogurt-like aroma and flavor and sweetness. In turn, juices inoculated with *Ln. pseudomesenteroides* were characterized by a spicy and sparkling flavor. After fermentation by these bacteria, the pineapple character of the juice decreased significantly, which, together with other characteristics, contributed to the lower overall quality of the product vs. the unfermented juice [43]. Changes in the flavor have also been observed in fermented soybeans. The presence of lipid oxidation products (n-hexanal and pentanal) contributes to the characteristic beany aftertaste of fermented soybeans [59]. As shown for kimchi, the amount and type of bacteria present in the product has a significant effect on its properties. Kimchi exhibits its unique flavor and aroma when the amount of LAB approaches its maximum level. It was shown that fermented kimchi generally had a less intense odor and taste of fresh cabbage during fermentation [66]. In contrast, the appearance of acidic and moldy odor and the taste of fresh sourness and mold were reported [50]. Sensory evaluation of a fermented pea-oat protein blend compared to unfermented extrudate revealed intensification of flavor qualities after fermentation. A cereal flavor and bitter and sour taste appeared. Fermented, medicinal, citrus, and soapy aromas were noted as well. The interaction of microorganisms with the plant matrix during fermentation results in formation of flavor compounds. They enhance the overall taste profile of the product, but some of them, e.g., cereal or bitter flavors, may be perceived as negative or undesirable in an analogous meat product. The texture of the plant material changes during fermentation as well. Products based on a fermented pea-oat protein blend were rated as dry and easily disintegrating. The low sensory moisture content of the fermented product obtained from pea and oat was confirmed by its low water holding capacity [67]. In fermented bio-yoghurt containing chickpea flour, the activity of LAB contributed to an increase in viscosity [68]. Lactic acid bacteria were also shown to ferment chia dough, improving its overall characteristics. After 24-h fermentation by *L. plantarum*, a decrease in consistency and increased viscosity were detected [69]. Viscosity as well as color change of the product was also observed in fermented prickly pear. After fermentation, the viscosity values decreased significantly compared to the raw plant material [70]. This tendency changed during storage when a significant increase in viscosity was observed in products fermented by LAB. Fermentation with the selected bacteria, especially with *Ln. mesenteroides*, additionally resulted in higher lightness values compared to raw prickly pear fruit [52]. In turn, the fermentation of quinoa flour had a positive effect on the texture and sensory properties of white bread based on this flour. The hardness of quinoa sourdough bread was lower than in the case of sourdough bread made from raw flour. This product also showed greater elasticity. Taste differences related to quinoa fermentation were observed as well. Fermentation of sourdough with quinoa resulted in a sour taste and aroma. The resulting bread was also more salty, probably due to the effect of acidification and proteolysis by LAB [60]. Studies have also shown that fermentation affects the rheological properties of soy-based gels. Gelation of soy proteins depends on various factors, including the ratio of globulins in the protein–polysaccharide mixtures and the molecular weight of polysaccharides or bacterial strains used for fermentation. Higher viscosity was observed in products fermented by combinations of *L. plantarum* and *Lactobacillus acidophilus* than in fermentation by a single strain.

Thus, a number of transformations take place in plant material fermented by bacteria. These include conversion of the chemical constituents of the plant matrix, resulting from the activity of enzymes of bacterial origin. The chemical composition of the plant matrix determines not only the physicochemical properties of the final product but also the range of microorganisms that will be able to grow in the material and carry out the fermentation process. However, the structure and abundance of the microbial community in the fermented plant product changes during the process. This is related to the compounds produced by the microorganisms and changes in other parameters, such as temperature. This is important since the composition of the microbial community defines the range of chemical compounds that will be formed during fermentation. The chemicals produced during fermentation of plant components, including volatile and bioactive compounds, influence the sensory and health-promoting properties of the final product. They also influence the textural and rheological properties of the plant matrix. All these factors contribute to the final characteristics of the product. Therefore, it is important to understand the determinants of the final outcome of the plant fermentation process in order to obtain, with strict control of the process conditions, a product with desired organoleptic, nutritional, and health-promoting properties and containing only beneficial microorganisms.

### 2.3. Health Benefits of Fermented Plants

**Figure 2:**
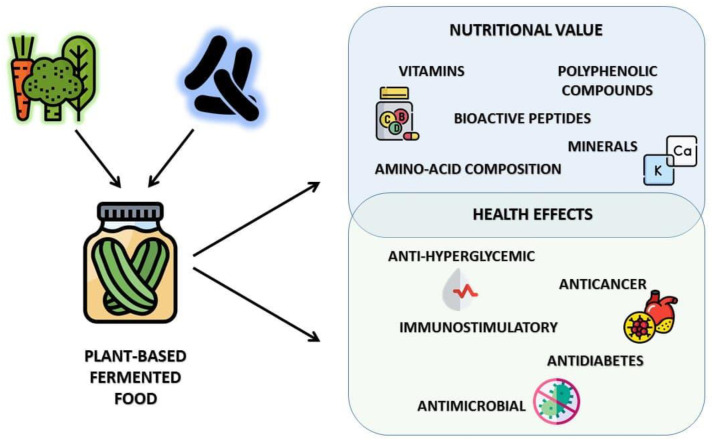
Beneficial effects of plant based fermented food.

Plant raw materials are a rich source of compounds with potential health-promoting properties. Depending on the food matrix type, they may have high content of protein, dietary fibers, polyphenolic compounds, and some microelements such as vitamins or minerals. As indicated in the literature, the presence of bioactive compounds in food products may result in their antioxidant [12,71,72], antimicrobial [73], antithrombotic [74], immunostimulatory, anticancer [75], and blood-glucose lowering properties [76] (Figure 2).

In plant material that undergoes the fermentation process, the action of enzymes and activity of microorganisms may induce changes in the nutritional properties and bioactive compound content, compared to the raw substrate [77,78]. A study on the cabbage fermentation process (kimchi production) conducted by Jung et al. showed that lactic acid bacteria utilizing sugar contained in the raw material produce organic acids and other fermentation products, which increase the taste and health-promoting qualities of the final product [50]. The fermentation of such cereals as wheat, barley, rye, and pseudo-grain amaranth also yields bioactive peptides and antimicrobial substances. Furthermore, during cereal fermentation, LAB improve the bioavailability of calcium, iron, and zinc. Through metabolism of starch contained in the plant material, they also produce essential short-chain fatty acids and other low-molecular-weight organic components, such as vitamins B and amino acids [56]. Many papers have reported changes in the content of polyphenolic compounds in fermented plant products induced by the presence of microorganisms. Studies on the effect of fermentation with the use of autochtonous microbial starters on the phenolic composition of olives have shown that fermentation of this plant material can produce up to a doubled quantity of polyphenols. Such phenolics as hydroxytyrosol, quercetin, luteolin, tyrosol, verbascoside, hydroxytyrosol acetate, and cyanidin-3-glucoside were identified in fermented olives [79]. Elevated total phenolic concentrations were also observed in cooked quinoa and buckwheat seeds fermented with indigenous lactic acid bacteria (*Pediococcus pentosaceus* and *Lacticaseibacillus paracasei*) [80]. An increase in total phenols (by 25%) was also observed in fermented chia dough, and their composition was strongly modified after 24-h fermentation by *L. plantarum*. Chlorogenic acid was found only in the fermented dough, whereas ferulic acid was detected in the non-fermented and fermented chia sourdough, with a significantly higher quantity in the latter [69]. It has been shown that an increase in phenolic compounds and soluble fiber is also induced by the enzymatic activity of LAB during sourdough fermentation of wheat and non-wheat flour. The high content of phenolic compounds has beneficial effects on hypertension. Several studies have shown that consumption of fermented apple juice lowers blood pressure and alleviates endothelial dysfunction [81,82]. Changes in the content of phenolic compounds directly influence the antioxidant properties of the food matrix, which have preventive effects against many diseases [54]. In general, fermented foods are characterized by higher antioxidant activity than raw products. Antioxidant activity was determined in a pear, apple, and carrot beverage fermented by two strains of *L. plantarum*. The authors observed an increase in the antioxidant activity with a maximum after four to eight days of fermentation [83]. Hwang et al. conducted a study on the effect of the fermentation process on the properties of hydroponic ginseng [74]. They used *Ln. mesenteroides* KCCM 12,010 P in the fermentation process. In the samples, the total phenolic compound content increased by 107.19% and the flavonoid content increased by 645.59%, compared to the control. The increase in the content of phenolic compounds was accompanied by enhancement of the antioxidant activity. In addition, the study indicated that fermented hydroponic ginseng exhibited anti-inflammatory and anti-adipogenic activity. After lipopolysaccharide stimulation, RAW 264.7 cells were characterized by lower content and expression of inducible nitric oxide synthase (iNOS), tumor necrosis factor-α (TNF-α), and interleukins (IL-1β and IL-6) after treatment with fermented samples. In parallel, reduced lipid accumulation in 3T3-L1 adipocytes was observed. Similarly, *Diospyros lotus* fruit fermented by *Microbacterium flavum* was characterized by higher content of several phenols, such as catechic, ellagic, and tannic acids, compared to the non-fermented matrix. The antioxidant activity of the product was confirmed by determination of the radical scavenging ability (DPPH and ABTS methods). Moreover, up to four-fold higher anti-α-glucosidase activity was noted. This indicates that fermented *D. lotus* has antioxidant and hypoglycemic properties [84]. Polyphenols in fermented apple juice had an inhibitory effect on the activity of starch digesting enzymes such as α-amylase and α-glucosidase in an animal model. This indicates their ability to control hyperglycemia [85]. Similarly, *Myrciaria dubia Mc. Vaugh* (camu-camu) fermented with *L. plantarum* and *L. helveticus* showed significantly reduced α-amylase and α-glucosidase activities. Inhibition of angiotensin-converting enzyme (ACE) activity was also reported in camu-camu fermented by *L. plantarum*, which suggests a beneficial effect of this product on treatment of type 2 diabetes. The hypoglycemic and hepatoprotective properties of fermented plant foods have also been demonstrated in studies on diabetic rats fed fermented noni juice. Diabetic rats receiving fermented juice showed reduced fatty degeneration of hepatocytes. These properties are probably closely correlated with the antioxidant activity and polyphenol content in fermented noni juice [54]. Furthermore, consumption of apple cider vinegar (ACV) improved the cellular response to oxidative stress. Rats with high cholesterol were administered ACV (1 mL/day) for one week, which increased the activity of key antioxidant enzymes such as catalase (CAT), superoxide dismutase (SOD), and glutathione peroxidase (GSH-Px) by 15–66% [86].

Some vitamins can only be obtained from foods of animal origin. The increase in the human population and the growing number of vegetarians and vegans necessitate a search for alternative sources of these compounds. Fortified or fermented foods may be a good solution to this problem. The deficiency of vitamins B can have serious health consequences. Adequate folate (B9) levels in the human organism are important not only in pregnant women. Deficiencies can be associated with serious health disorders such as Alzheimer’s disease, osteoporosis, coronary artery disease, and colon cancer. Some research has indicated that LAB can increase the folate content in food. Injera is a type of fermented bread consumed in Ethiopia. The fermentation process is mainly carried out by LAB. Studies conducted by Tamene et al. indicated that some of the strains isolated from injera produced from 1 to 43 μg/L of folates [87]. *L. plantarum* species (P2R3FA) showed the highest ability to produce these compounds. Moreover, a study on vitamin B9-deficient rats showed that folate levels increased after the animals consumed feed supplemented with this strain. Bacterial activity in fermented foods can also result in the synthesis of vitamin B12. Several *Propinobacterium* strains and LAB are the main producers of this vitamin. A significant increase in vitamin B12 content (up to 0.97 μg/100 g) was obtained by fermenting lupine using *Rhizopus oryzae* spores along with *Propionibacterium freudenreichii* [88]. Similarly, an increase in vitamin B12 content was observed in fermented cauliflower and a bean mixture. The presence of *L. plantatrum* strain 299 in the food matrix resulted in a 66% increase in the content of this vitamin [89]. Available literature data also indicate an increase in riboflavin (B2) and vitamin K or C in fermented plant foods. An increase in vitamin content in the food matrix is associated with an increase in antioxidant and immunostimulatory activity [1,90].

Analyses of fermented bamboo shoots have shown that the fermentation process can also lead to elevated quantities of certain minerals (calcium, sodium, magnesium, iron, and sulfur) in the final product [91]. It has been suggested that the increase in the mineral content is caused by reduction in dry matter as a result of carbohydrate and protein decomposition by microorganisms during fermentation of the plant material [92]. Research on fermented dough containing flour from legumes or pseudocereals has shown that this process can improve nutritional values. The addition of legume flours to fermented bread sourdough resulted in increased concentrations of free amino acids and gamma-aminobutyrate (GABA), enhanced phytase activity, and improved protein digestibility [13]. Generation of functional molecules was also observed when *L. plantarum* was used to ferment date palm fruit puree (*Phoenix dactylifera* L.). In these experiments, fermentation of raw plant material yielded GABA acid, dietary fiber, and conjugated fatty acids [52]. GABA was also generated by fermentation of cactus cladodes (*Opuntia ficus-indica* L.) pulp by *Levilactobacillus brevis* POM2 and POM4 [93]. GABA is an exogenous amino acid synthesized by certain strains of lactic acid bacteria. It acts as an inhibitory neurotransmitter in the central nervous system. It can have antidepressant and anxiolytic effects on the human organism and can regulate hormone secretion [54]. The presence of GABA has also been linked to antioxidant and antidiabetic effects and reduction of blood pressure. During fermentation, higher levels of peptides and free amino acids are observed in fermented foods due to the proteolytic activity of such bacteria as LAB. Cystine, histidine, and asparagine have been detected in fermented soymilk [1]. In fermented cereal products, matrix proteins are converted into antihypertensive bioactive peptides through the proteolytic activity of bacteria [94].

Fermented soy-based foods have also been found to produce bioactive peptides with antidiabetic and ACE inhibitory effects [76]. Arulrajah et al. conducted a study on the properties of kenaf seed proteins fermented by *Lacticaseibacillus casei* [95]. After the fermentation process, the proteins were fractionated and peptides were identified. The product showed antibacterial activity against *Salmonella typhimurim*, *Escherichia coli*, *Pseudomonas aeruginosa*, *Staphylococcus aureus*, *Bacillus subtilis*, and *Streptococcus pyogenes*. The MIC value was estimated at 4 mg/mL for all tested strains.

In addition, FODMAPs (Fermentable oligosaccharides, disaccharides, monosaccharides, and polyols) consumed in food can cause symptoms of irritable bowel syndrome (IBS). Their presence can be reduced in such cereal products as wheat bread by bioprocessing with endogenous enzymes and microbial fermentation [1]. Usually, the ability to reduce FODMAPs in fermentation processes of cereal products is exhibited by yeast cells. However, some bacteria have a positive effect on reducing these compounds as well [96]. Lactic acid bacteria, e.g., *L. plantarum* and *Latilactobacillus curvatus* strains isolated from rye sourdough, were capable of fermenting fructose, mannitol, and sorbitol [97]. The metabolism of LAB growing on plant matrices often results in the synthesis of polyols. The type of sugars contained in plant materials has been shown to affect the efficiency of polyol synthesis and the growth rate of FLAB (such as *Fructilactobacillus florum* and *Fructobacillus tropaeoli*) [96]. The production of phenolic compounds by lactic acid bacteria during fermentation of plant matrices was also observed in many other studies recently reviewed by Filannino et al. [17]. Changes in the FODMAP level were also reported during wheat dough fermentation with FLAB. Albiac et al. indicated the ability of commercial enzymes and metabolically active FLAB to hydrolyze fructans and deplete fructose during fermentation [98]. The authors selected *Apilactobacillus kunkeei* B23I and *Fructobacillus fructosus* MBIII5 as a potential culture starter for bakery products with low FODMAP content. Interesting changes were also observed in pineapple juice fermented by *W. cibaria.* During fermentation, a significant reduction in the concentration of glucose was observed in the material, which was utilized by the bacteria. The amount of sucrose in the material was also decreased; hence, the final product contained 20% lower amounts of sugars than the unfermented juice [43]. A significant decrease in sugars (glucose and fructose) was also observed during fermentation of prickly pear. In this study, the fermentation bacteria utilized citric acid contained in the plant material as well [52].

Due to the presence of microorganisms, fermented products may have potential probiotic properties supporting the human immune system. Furthermore, plant-based fermented foods are good carriers of probiotics and prebiotics [99]. As indicated in the literature, fermented table olives can be a good source of probiotic bacteria. Montoro et al. isolated 31 strains of *Lactiplantibacillus pentosus* from naturally fermented green Aloreña table olives [100]. The selected strains were tested for their probiotic properties and the results indicate their ability to grow and survive in stimulated gastrointestinal conditions. In addition, efficient auto-aggregation with host cells and co-aggregation with such pathogenic bacteria as *Staphylococcus aureus* and *Listeria innocua* were demonstrated, which is a good defense against intestinal and food pathogens [100]. A study conducted on a model organism (*Caenorhabditis elegans*) previously fed isolated *Lactobacillus* strains from naturally fermented olives indicated that *L. pentosus* and *Loigolactobacillus coryniformis* strains induced a prolonged effect and protected against pathogen-induced infections. In addition, these strains showed adhesion to human Caco-2 intestinal epithelial cells and competed for cell adhesion with foodborne pathogens [101]. In addition to their antimicrobial properties, fermented probiotic products may have a positive effect on reducing obesity and body fat. A study conducted on mice fed fermented cactus pear juice indicated a significant decrease in body weight and in the adipose index [102]. Besides fermented fruits and vegetables, fermented legume beverages are potential carriers of probiotics. For example, soy milk fermented by *L. casei* favorably modulated the intestinal microbiota [103], while chickpeas showed high probiotic viability after fermentation [104]. Fermented cereal foods can also be good sources of probiotic bacteria. LAB strains isolated from wheat bran sourdough were tested for antifungal and probiotic activity. The results indicate that the isolated strain *Pediococcus pentosaceus* (CE65) is characterized by antifungal activity and is able to produce exopolysaccharides. Additionally, it can be potentially used as a probiotic strain [105]. In addition to improving the rheological properties of products, exopolysaccharides (EPS) produced by some bacteria have antioxidant, antimicrobial, immunomodulatory, and antitumor properties. Additionally, they facilitate colonization of the intestine by probiotic bacteria through cell adhesion [54]. EPS produced by *L. plantarum* BR2 exhibited antioxidant properties and, applied at various concentrations, decreased α-amylase and α-glucosidase activities, indicating antidiabetic potential. Furthermore, its application in vitro at a concentration of 0.1% resulted in a 45% reduction in cholesterol levels, with no cytotoxicity against normal H9C2 cells [106]. Fermented plant-based food can also be a good source of EPS. Fermented wholemeal milk from quinoa contained 40 mg/L EPS produced by *W. cibaria* MG1. High concentrations of EPS (dextran) also improved the texture of the product [107]. In turn, *Ln. pseudomesenteroides* DSM 20,193 used for fermenting fava bean flour had a high capability of EPS synthesis [108]. EPS produced by starter cultures in fermented products provide protection against pathogens and toxins, since these compounds can bind to the intestinal wall, thereby preventing pathogen adherence. The main studies that have been conducted to date on plant-based fermented food and their health benefits are summarized in Table 2.

Health problems related to lifestyle diseases, which are increasingly common in society, determine the search for preventive solutions. With their confirmed antimicrobial, immunomodulatory, antidiabetic, and anticancer properties, fermented plant foods seem to be a good component of everyday diet. Moreover, they can be a good carrier of probiotics. The enrichment of the diet with probiotics is associated with numerous health benefits such as the prevention and treatment of diarrhea, alleviation of lactose intolerance symptoms, and modulation of the intestinal microbiota. Dairy products are still the main source of probiotic bacteria; however, as indicated in the literature, fermented plant foods can also be good matrices for probiotics. They are also a good alternative for subjects with allergies who cannot eat dairy products or for vegetarians and vegans, providing them with basic nutrients. The consumption of fermented products satisfies the daily needs for basic nutrients. However, more in vivo and in vitro research is needed to elucidate the mechanisms of interactions between the food matrix and the organism.

## 3. Conclusions

Fermented plant products have been produced for thousands of years worldwide. They play a pivotal role in the diet for humans, who do not necessarily have knowledge of the complexity of microbial cultures. Nowadays, it is possible to control microbial populations by allowing beneficial microbes to multiply and inhibiting the growth of spoilage microbes, thus modifying the functional and health properties of fermented foods. The presence of a group of desirable microorganisms ensures success in food preservation owing to reduction of pH, production of organic acids, and decomposition of complex carbohydrates into bioactive compounds. Fermentation based on plant material is a virtually inexhaustible supply of dynamic microbial communities that utilize complex plant compounds and create a new chemical profile of the product, often with health-promoting properties. Although the occurrence of LAB is widely known, the information about their specific ecological niches, genetics, physiological characteristics, and impact on human health is constantly expanding. Recent discoveries, especially in the area of genomics and probiotic potential, lead to the development of high-quality nutritious and safe food.

## Figures and Tables

**Table 1:** Summary of key molecular features responsible for bacterial adaptation to the niche of fermented plant foods.

Species	Main Characteristics of Molecular Adaptation	Literature
*Lactiplantibacillus plantarum*	One of the largest genomes (3.5 Mb on average), The “universal” genome allows adaptation to a wide range of environments. Each strain has an individual sugar metabolism profile, but the genes are grouped together in the genome (so-called island). Presence of genes encoding amylose and starch degradation Genes dltA&D, gadB, and clpL responsible for acid tolerance and plantaricin genes Gene encoding a phosphonate ABC transporter increasing the trapping of phosphate, when its concentrations are low in the fermented vegetable environment	[18,39,40,41]
*Latilactobacillus sakei*	Ability to conduct a homo- and heterolactic fermentation Genes for survival on residual sugar sources in the final stages of kimchi feremntation	[19,20,21]
*Latilactobacillus curvatus*	Plant-derived strains have more genes encoding sugar metabolism and transporters than strains isolated from other environments	[23]
*Lactococcus* spp.	“Domesticated” and “environmental” genomes can be distinguished; those used in industry are poorer in metabolism genes Strains associated with a plant niche use a wider range of metabolic pathways than dairy strains, since lactose is the main carbon source in milk, while each plant niche has an individual carbohydrate composition. Genes responsible for biofilm formation	[26,27]
*Lactococcus lactis*	Pathway related to the metabolism of plant sugars raffinose and arabinose	[26,28]
*Apilactobacillus micheneri*	Fructophilic lactic acid bacteria Reductive evolution of fructophilic genomes, adaptation to high concentrations of simple sugars: glucose and fructose, which are prevalent in the fermented fruit environment The bacteria need an external electron acceptor to balance NAD/NADH	[33,35,42]
*Leuconostoc mesenteroides*	Ability to grow at relatively low temperatures A frameshift mutation within the atpC gene and overexpression of F0F1 ATPase may contribute to cancelling the lethal effects of acidification during fermentation. Mannitol dehydrogenase converting fructose present in early kimchi or sauerkraut fermentation. Mannitol is considered a significant ingredient responsible for the specific flavor of fermented vegetables. Intensive fermentation of glucose in the initial stages of fermentation followed by fructose, mannose trehalose, and sucrose	[19,36]
*Weissela koreensis*	Reductive evolution of genomes Dominance in the later stages of kimchi fermentation due to the ability to utilize ribose, mannose, arabinose, and gluconate	[20,43]
*Enterococcus faecium*	Adaptation to soybean, fructose, and mannose metabolism Missing genes of virulence factors, genes responsible for antibiotic resistance, and mobile elements	[38]

**Table 2:** Summary of plant-based fermented foods and their health effects.

Fermented Products	Health Effects	Literature
Apple juice	Reduction of blood pressure	[81,82]
Apple juice	Hyperglycemia control	[82]
Noni juice	Hypoglycemic and hepatoprotective properties	[54]
Pear, apple, and carrot beverage	Increased antioxidant activity	[83]
Apple cider vinegar	Improved cellular response to oxidative stress	[86]
Hydroponic ginseng	Increased antioxidant, anti-inflammatory, and anti-adipogenic activity	[71]
*Diospyros lotus* fruit	Antioxidant activity and hypoglycemic properties	[84]
*Myrciaria dubia Mc. Vaugh* (camu-camu)	Effect in type 2 diabetes	[45]
Lupine	Increased B12 content	[88]
Injera	Increased folate content	[87]
Cauliflower	Increased B12 content	[89]
Bamboo shoots	Elevated quantities of certain minerals	[91]
Kenaf seed proteins	Antibacterial activity	[95]
Wheat dough	Reduction of FODMAP content	[98]
Pineapple juice		[43]
Prickly pear		[52]
Table olives	Probiotic properties	[100]
Soy milk		[103]
Chickpeas		[104]
Cactus pear juice	Probiotic properties, reduction of obesity	[102]
Wheat bran sourdough	Probiotic properties, antifungal activity	[105]

## Data Availability

The analyzed publications are available from the authors.
